# Case reports: Intraoperative migratory retinal venous thrombus in proliferative diabetic retinopathy

**DOI:** 10.3389/fmed.2024.1372831

**Published:** 2024-09-09

**Authors:** Danni Lyu, Huan Liu, Yijiong Fang, Yao Wang

**Affiliations:** ^1^Eye Center of the Second Affiliated Hospital of Zhejiang University School of Medicine, Hangzhou, Zhejiang, China; ^2^Department of Ophthalmology, The First People’s Hospital of Lin’an District, Hangzhou, Zhejiang, China; ^3^Department of Ophthalmology, The First People’s Hospital of Xiaoshan District, Hangzhou, Zhejiang, China

**Keywords:** fibrovascular membrane delamination, migratory retinal venous thrombus, proliferative diabetic retinopathy, tractional retinal detachment, vitrectomy

## Abstract

**Purpose:**

This study aimed to study the characteristics, possible causes, and clinical implications of intraoperative migratory retinal venous thrombus in proliferative diabetic retinopathy (PDR).

**Cases:**

Two middle-aged Chinese patients with diabetes mellitus presented with blurred vision and were diagnosed with PDR and tractional retinal detachment (TRD). An interesting phenomenon was observed during pars plana vitrectomy in both patients. Movement of tiny white thrombi and interruption of blood flow were observed in a branch of the central retinal vein when the vein was pulled at the time of fibrovascular membrane delamination and disappeared with the elimination of retinal traction after finishing the process of delamination. Laboratory studies revealed abnormal erythrocyte sedimentation rate, fibrinogen, D-dimer, international normalized ratio, and IgA anti-β2-glycoprotein I in one patient and elevated fibrinogen and IgA anticardiolipin in the other. Follow-up examinations at 1 week, 1, 3, and 6 months postoperatively showed good prognosis. Fluorescein fundus angiography at 1 month postoperatively showed neither embolus sign nor prolonged venous filling time in both patients.

**Discussion:**

Local blood stasis of the retinal vein persistently dragged by the fibrovascular membrane may result in thrombogenesis, and traction of the retina during the delamination process may lead to the movement of thrombi. On the other hand, endothelial injury and disordered local blood stasis during delamination may also activate the biological coagulation process and instant thrombus formation. As well, antiphospholipid antibodies may also be a risk factor of ocular thrombogenesis.

**Conclusion:**

This study provides the first videos recording migratory thrombus in terminal vessels, which indicates that fibrovascular membrane in PDR can lead to thrombogenesis due to dragging and hemostasis of the involved retinal vein. PDR patients with fibrovascular membranes may benefit from early relief of vascular traction through fibrovascular membrane delamination.

## Introduction

Diabetic retinopathy (DR) is a prevalent and severe complication of diabetes mellitus (DM) (1). Similar to the development of DM-associated complications in other systems, the disturbance of microcirculation is a notable pathological change in DR (2, 3). Proliferative diabetic retinopathy (PDR) leads to profound vision impairment due to severe complications including vitreous hemorrhage and tractional retinal detachment (TRD). For cases of PDR accompanied by TRD, pars plana vitrectomy (PPV) is necessary. This study is the first to present imaging evidence of an interesting phenomenon observed during PPV and fibrovascular membrane delamination for PDR: migratory retinal vein thrombosis. The article analyzes and discusses the potential causes and clinical implications of this phenomenon, aiming to reach a better understanding of DR and the timing of surgical interventions for PDR with concurrent TRD.

## Case description

**Figure 1:**
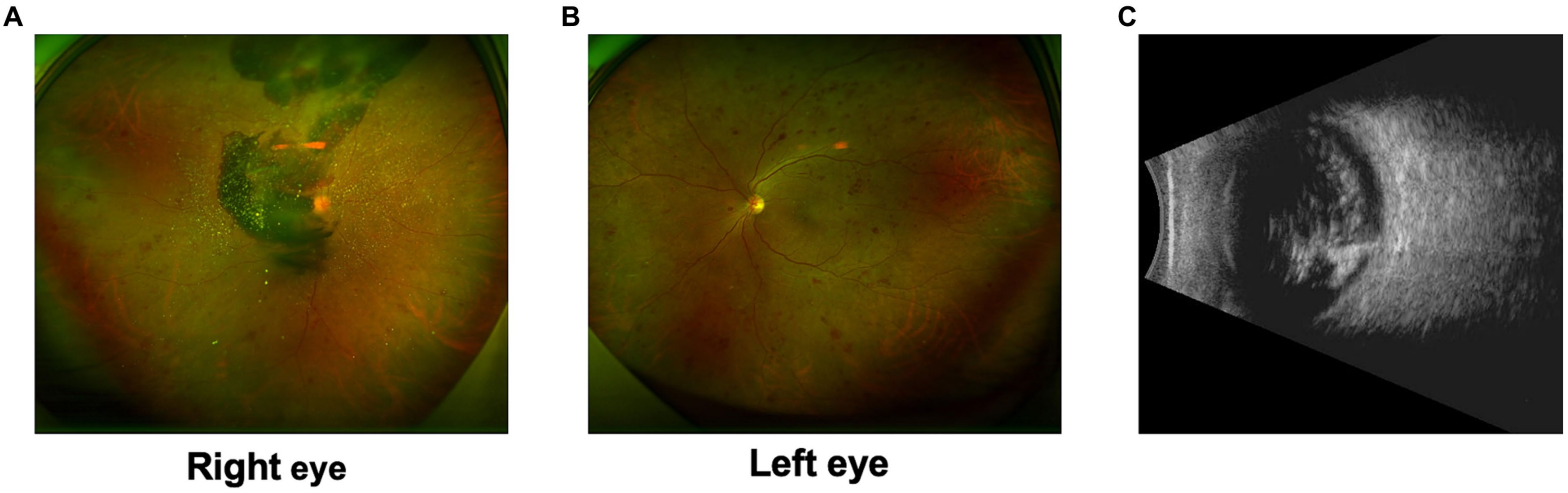
Preoperative examination of Patient 1. **(A)** Ultra-widefield fundus photography of the right eye revealed vast preretinal hemorrhage in the posterior polar and superior regions, together with scattered intraretinal hemorrhage spots and asteroid hyalosis in the periphery. **(B)** Ultra-widefield fundus photography of the left eye revealed microaneurysms and retinal hemorrhage in all quadrants of the fundus as well as a few hard exudates in the posterior polar retina. **(C)** Ultrasound of the right eye revealed vitreous opacity and tractional retinal detachment.

A 54-year-old Chinese woman (Patient 1) presented to our ophthalmology clinic with blurred vision in her right eye for over a month. The patient had a 7-year history of type 2 diabetes mellitus (T2DM) with poor control of glucose level. She had phacoemulsification cataract extraction and intraocular lens (IOL) implantation in both eyes 1 year ago. At presentation, her best-corrected visual acuity (BCVA) was finger count in the right eye and 20/50 in the left eye. Fundus examination of the right eye revealed vast preretinal hemorrhage in the posterior polar and superior regions, together with scattered intraretinal hemorrhage spots and asteroid hyalosis in the periphery (Figure 1A). Microaneurysms and retinal hemorrhage in all quadrants of the fundus as well as a few hard exudates in the posterior polar retina were observed in the left eye (Figure 1B). Ultrasound revealed vitreous opacity and tractional retinal detachment (TRD) in the right eye (Figure 1C). She was diagnosed with proliferative diabetic retinopathy (PDR) in both eyes. Laboratory studies revealed elevated erythrocyte sedimentation rate, fibrinogen, and D-dimer, as well as a reduced international normalized ratio. IgA anti-β2-glycoprotein I (β2-GPI) remained positive during a follow-up over 12 weeks since the first visit. At admission, fasting blood glucose (FBG) and postprandial blood glucose (PBG) were 5.53 mmol/L and 16.60 mmol/L, respectively. Blood pressure was 160/100 mmHg. Glycosylated hemoglobin HbA1c was 8.2%.

A 23-gauge PPV was performed for the patient using a standard vitrectomy system (Stellaris Elite™ PC, Bausch & Lomb Inc., USA). Valved cannulas were selected to allow stable intraoperative fluidics and intraocular pressure controlled approximately 30 mmHg. Resight 700 Fundus Viewing System (Carl Zeiss Meditec AG, Germany) with a spheric widefield lens 128D and an aspheric lens 60D was used. During the procedure, preretinal hemorrhage in the posterior polar and superior regions, scatted hard exudates, as well as fibrovascular membrane and local TRD along the superotemporal retinal vessel arcade, were observed. In brief, core vitrectomy and posterior vitreous detachment (PVD) were performed, and preretinal hemorrhage was eliminated. An unimanual segmentation-and-delamination approach and suck-and-cut technique were applied to remove the fibrovascular membrane. Forceps were also used to loosen and separate tightly adherent fibrovascular membranes, followed by cutting using the vitreous probe. Peripheral vitrectomy, panretinal photocoagulation (PRP), and air tamponade were performed afterward.

**Figure 2:**
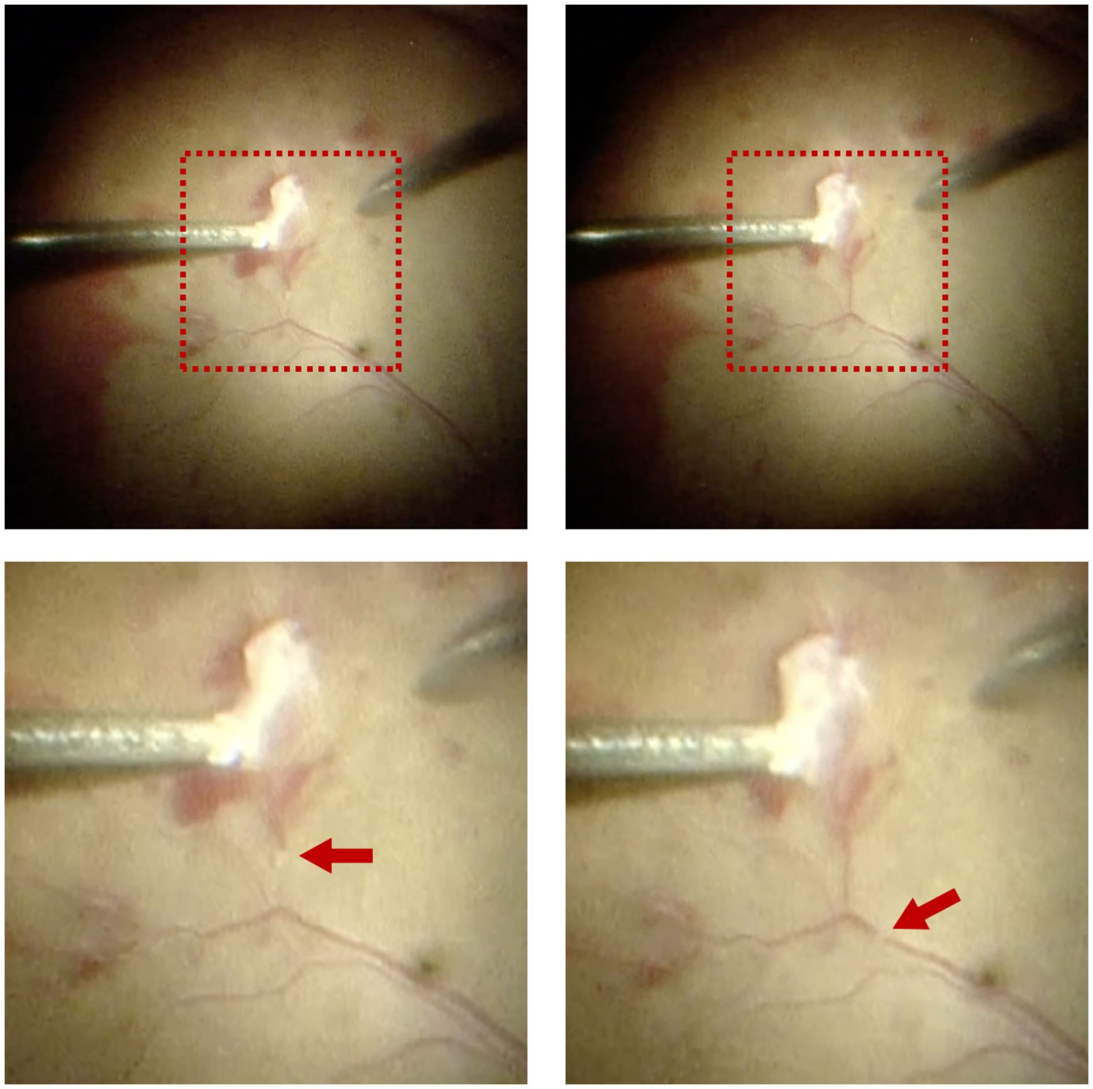
Intraoperative observation of migratory retinal venous thrombus in Patient 1. Rapid movement of tiny white thrombi was observed in the superotemporal branch of the central retinal vein at the time of fibrovascular membrane delamination. The upper two images displayed the full view, while the lower two images displayed the magnified view. Red arrows indicated the migratory retinal venous thrombus.

Interestingly, rapid movement of tiny white thrombi was observed in the superotemporal branch of the central retinal vein at the time of fibrovascular membrane delamination (shown in Figure 2 and Supplementary Video S1). The thrombi moved from the site of delamination toward the optic disc three times when the surgeon pulled the retinal vein during the process of delamination, with a duration of approximately 2–3 s each time. This phenomenon disappeared with the elimination of retinal traction after finishing the process of delamination.

**Figure 3:**
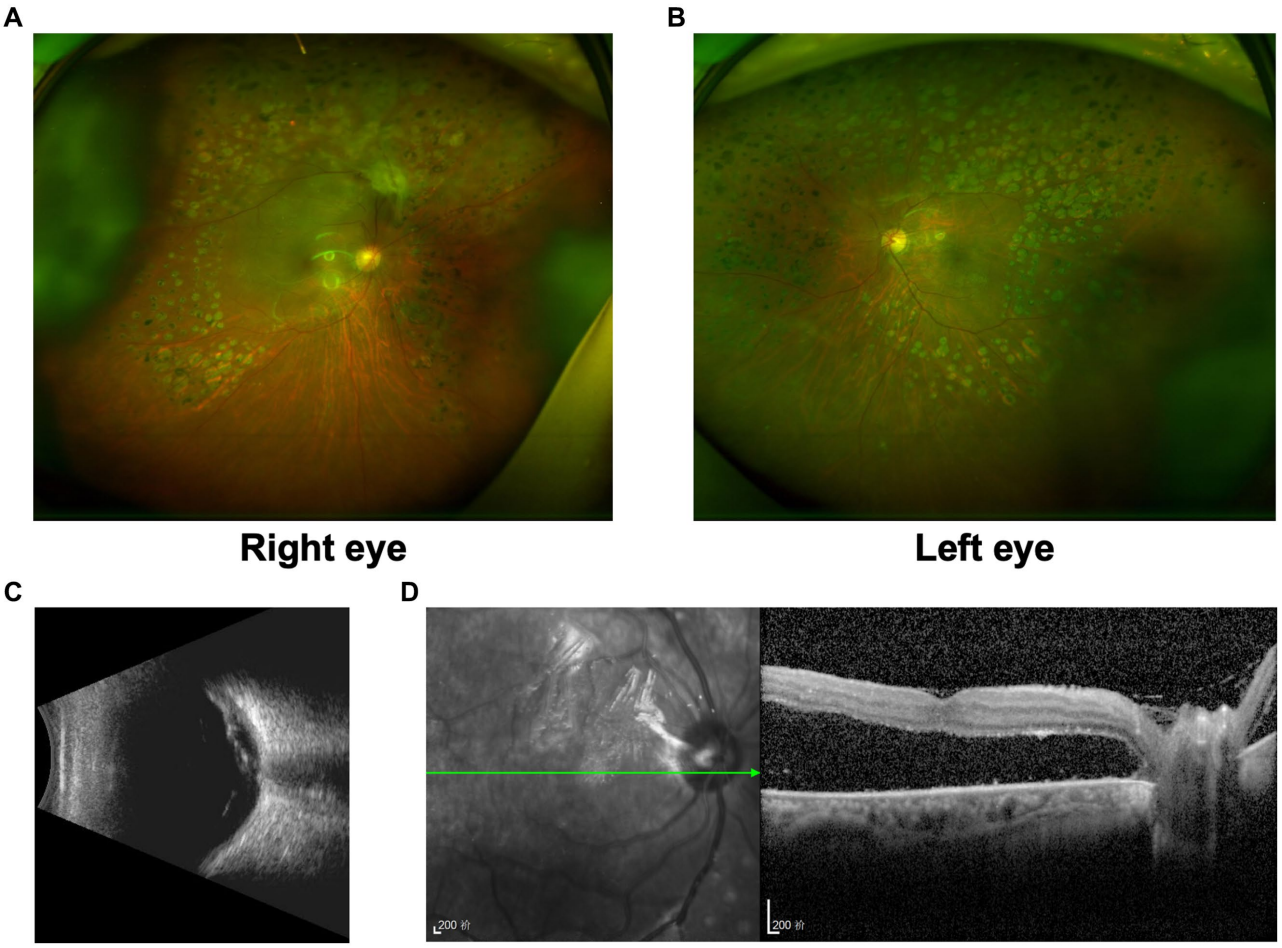
Preoperative examination of Patient 2. **(A)** Ultra-widefield fundus photography of the right eye revealed evident tractional retinal detachment with the involvement of the macular area, together with previous PRP spots in the periphery. **(B)** Ultra-widefield fundus photography of the left eye revealed previous PRP spots in the periphery. **(C)** Ultrasound of the right eye revealed evident tractional retinal detachment and slight hemorrhagic vitreous opacity. **(D)** OCT of the right eye revealed retinal detachment and epiretinal membrane in the macular area.

Similar phenomenon was also observed in another 63-year-old Chinese woman (Patient 2) who presented with decreased vision in the right eye for 2 months. She had phacoemulsification cataract extraction and IOL implantation in both eyes 10 years ago, and PRP in both eyes 8 years ago (Figures 3A,B). She had a history of T2DM with fair control of glucose levels for over 10 years. Her BCVA was 20/133 in the right eye and 20/25 in the left eye. Evident tractional retinal detachment was observed in the right eye, with the involvement of the macular area (Figures 3A,C). Slight hemorrhagic vitreous opacity was also present. OCT revealed retinal detachment and epiretinal membrane in the macular area (Figure 3D). At admission, FBG and PBG were 6.49 mmol/L and 11.80 mmol/L, respectively. Blood pressure was 154/77 mmHg.

Laboratory studies revealed positivity for IgA anticardiolipin (aCL) and elevation in fibrinogen. She was also diagnosed with PDR in both eyes, and a 23-gauge PPV was scheduled. During the surgery, a massive fibrovascular membrane was observed in the superior retina along the vessel arcade, leading to evident TRD in the superotemporal region and a small retinal hole in the superior region. Core vitrectomy and PVD were performed, followed by fibrovascular membrane delamination. Perfluorocarbon liquid was used to flatten the detached retina. Retinal photocoagulation was performed around the retinal hole and in the peripheral retina. Silicone oil tamponade was performed at the end of the surgery.

**Figure 4:**
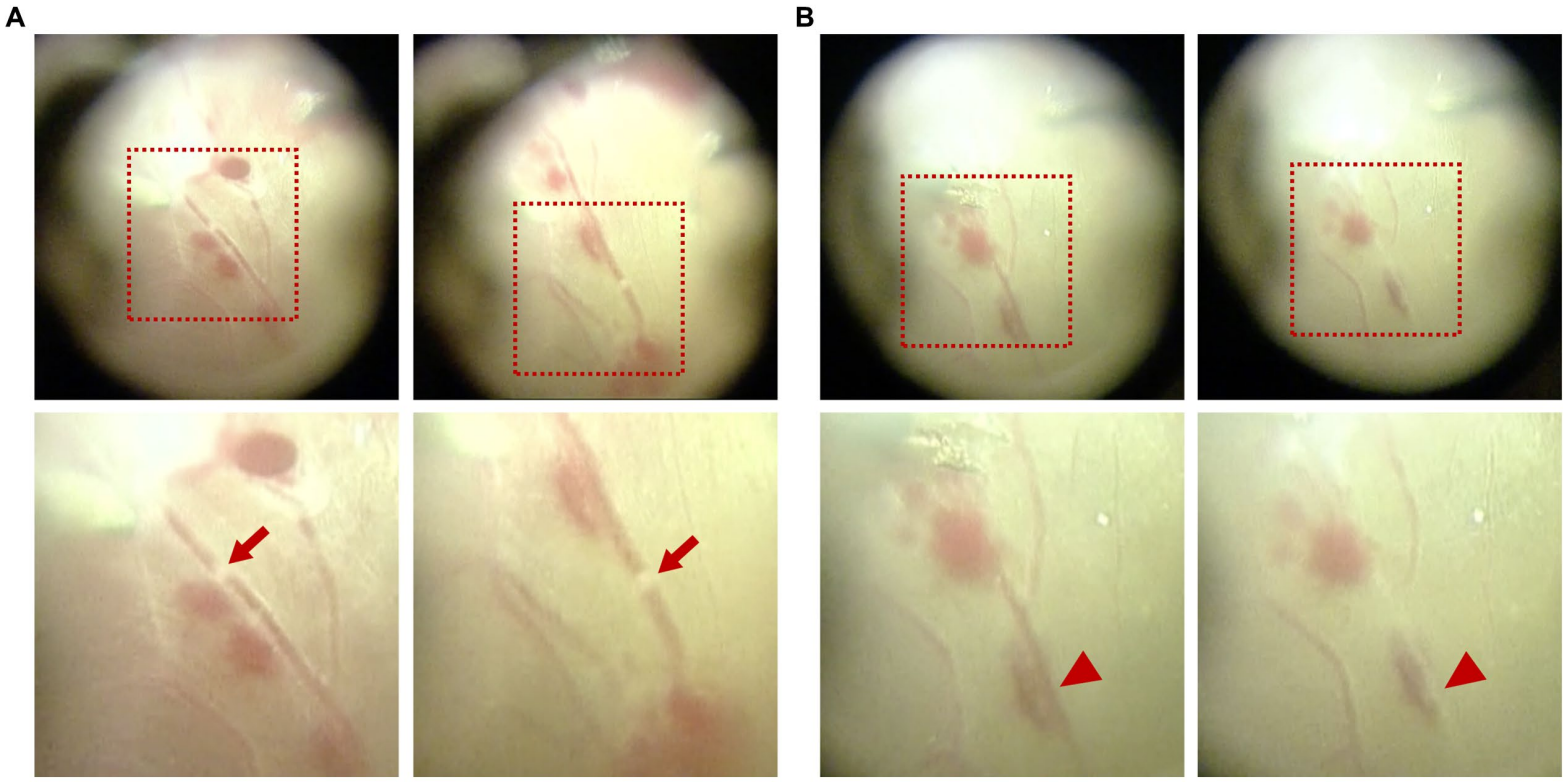
Intraoperative observation of migratory retinal venous thrombus and blood flow interruption in Patient 2. **(A)** White migratory thrombi with a similar diameter to the vessel were observed during fibrovascular membrane delamination. **(B)** Interruption of blood flow was observed with evident segmental whitening of the vein, which appeared during the process of membrane delamination. The upper images displayed the full view, while the lower images displayed the magnified view. Red arrows indicate the migratory retinal venous thrombus. Red arrowheads indicate blood flow interruption.

It is worth mentioning that white migratory thrombi with a similar diameter to the vessel were observed twice in a branch retinal vein during fibrovascular membrane delamination, with a duration of approximately 2–3 s each time, similar to that in Patient 1 (shown in Figure 4A and Supplementary Video S2). Interruption of blood flow was also observed for three times with evident segmental whitening of the vein, which appeared when the membrane delamination began and disappeared immediately once the delamination stopped (shown in Figure 4B and Supplementary Video S3).

**Figure 5:**
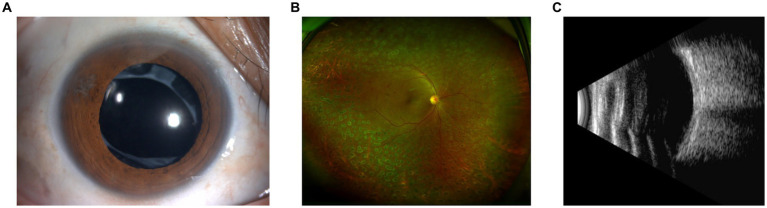
Postoperative examination of Patient 1 at 3 months. Anterior segment photography **(A)** revealed no positive findings. Ultra-widefield fundus photography **(B)** and ultrasound **(C)** revealed a well-attached retina.

**Figure 6:**
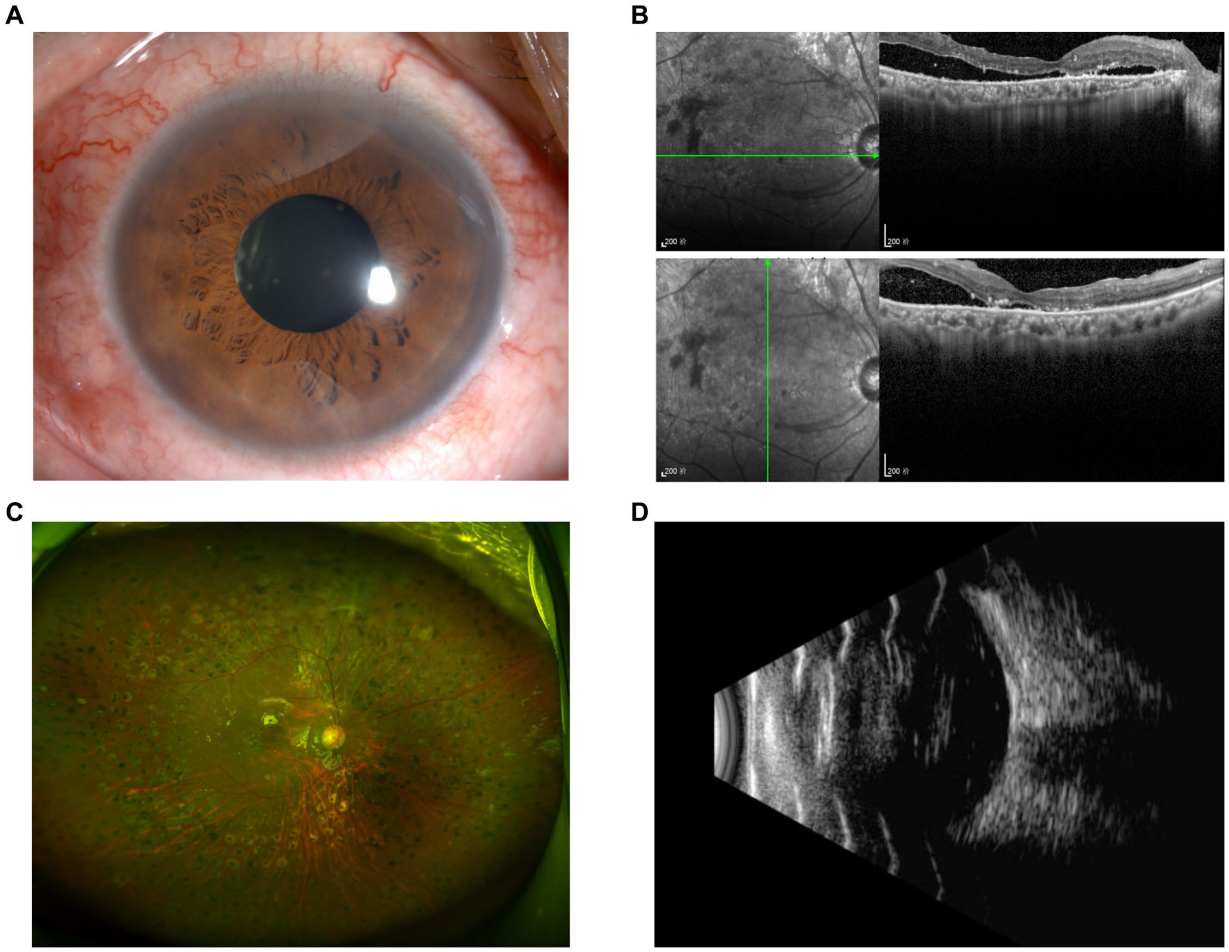
Postoperative examination of Patient 2 at 6 months. Anterior segment photography **(A)** revealed no positive findings. Slight local subretinal fluid was discovered by OCT **(B)**, yet no obvious retinal detachment was detected according to ultra-widefield fundus photography **(C)** and ultrasound **(D)**.

**Figure 7:**
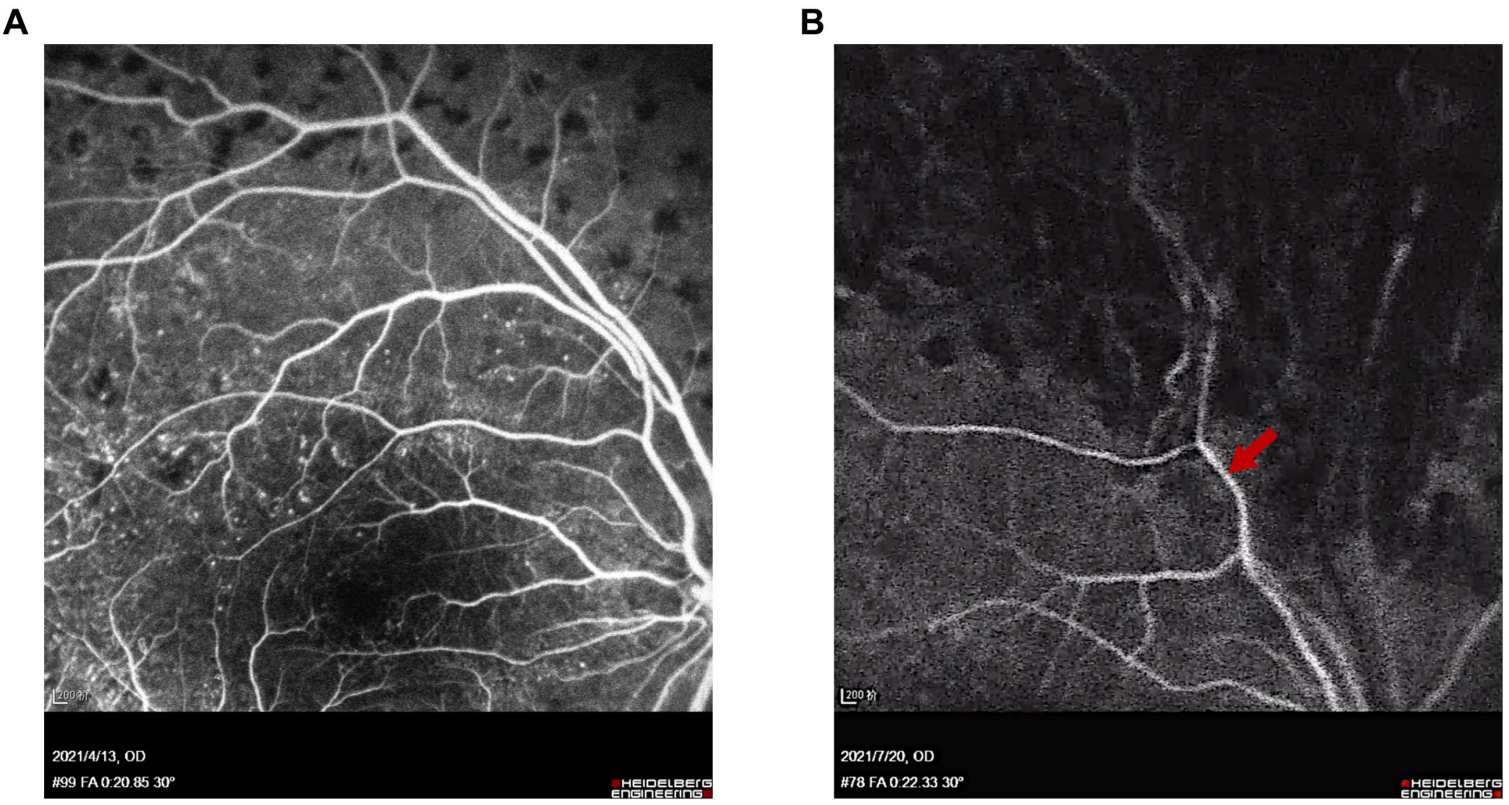
FFA of Patients 1 and 2 at 1 month after surgery. **(A)** Venous phase (at 20 s) of FFA showed normal venous filling and no embolus sign in Patient 1. **(B)** Arteriovenous phase (at 22 s) of FFA showed delayed venous filling without embolus sign in Patient 2. Red arrow indicates the location of the superotemporal branch of the central retinal vein.

Follow-up examinations were scheduled at 1 week, 1, 3, and 6 months postoperatively. Examinations of Patient 1 demonstrated a well-attached retina (Figure 5) and improved BCVA at 3 months after surgery. In Patient 2, though BCVA was much improved, optical coherence tomography showed persistent local detachment in the macular area. Silicone oil was removed at 4 months postoperatively after the local retinal detachment was mostly relieved. Follow-up at 6 months postoperatively revealed that the slight local subretinal fluid remained, yet no obvious retinal detachment was detected by ultrasound (Figure 6). Fluorescein fundus angiography (FFA) was carried out in both patients at 1 month postoperatively, which revealed normal venous filling without embolus sign in Patient 1 (Figure 7A and Supplementary Videos S4, S5) and delayed venous filling without embolus sign in Patient 2 (Figure 7B and Supplementary Videos S6, S7).

The study protocol adhered to the tenets of the Declaration of Helsinki and was approved by the Medical Ethics Committee of the Second Affiliated Hospital of Zhejiang University School of Medicine, Hangzhou, China. All the clinical data were obtained from the electronic medical record system, with the patients’ consent.

## Discussion

These two cases are presented for discussion of the possible causes and clinical implications of the thrombi observed during fibrovascular membrane delamination in PDR surgery.

Up till now, no similar phenomenon has been reported, to our knowledge. There are two probable assumptions. First, according to these two cases, the venous thrombus is more likely to occur in PDR accompanied by TRD, with significant traction and twisting of the retinal vein. A probable interpretation is that local blood stasis of the retinal vein persistently dragged by the fibrovascular membrane may result in thrombogenesis, and traction of the retina during the delamination process may lead to the movement of thrombi. Second, since there is no evident thrombus observed before delamination, the thrombi were more likely to be a result of instant biological coagulation activation due to endothelial injury and disordered local blood stasis during the delamination process. As we know, red thrombi composed mainly of erythrocytes rather than fibrin are more commonly seen in veins due to lower blood flow velocity (4). Yet in the condition of local venous stasis or turbulence of blood flow, mixed thrombi with a stratified body composed of a mixture of erythrocytes, fibrin, and platelets may also be slowly formed in veins. It has also been reported that the microthrombi in the retinal capillaries of DR individuals were identified to be fibrin-platelet thrombi (5). White-centered retinal hemorrhages, also known as Roth spots, are frequently observed in the case of inflammation (e.g., subacute bacterial endocarditis), malignancy (e.g., leukemia), and other systematic conditions such as anemia, DR, and hypertension. The formation of Roth spots has been demonstrated to be related to fibrin-platelet thrombi formation due to retinal microvascular and capillary injury, rupture, and hemorrhage, which may share some resemblance with the venous thrombi in our cases (6, 7). Blood status may recover after the removal of the fibrovascular membrane in time, which may explain the negative FFA finding in Patient 1 postoperatively after relief from traction and recovery of normal blood supply. However, in patients with more severe TRD, the blood flow stasis may not be completely reversible after prolonged traction of the fibrovascular membrane, as indicated by the FFA finding in Patient 2 postoperatively, which showed delayed venous filling.

Diabetic retinopathy (DR) is the most common vision-threatening disease among the working-age population, accounting for 2.5% of the 37 million blindness cases worldwide, according to the data from the World Health Organization (1). It is a common microvascular complication of diabetes, characterized by progressive microvascular injury contributing to retinal ischemia, neovascularization, and altered retinal permeability (8). The abnormality in the coagulation–fibrinolytic system is one of the main pathological alterations in DR, especially in PDR, which leads to retinal ischemia (9, 10). Increased prevalence of microthrombi has been reported in retinal microvasculature in both human and animal models with diabetes (5, 11), though no retinal venous thrombus has been directly observed so far. Fujisawa et al. found an independent association between T2DM and increased blood viscosity due to high fibrinogen levels, which in turn play a role in the advancement in DR (12). Patients with PDR had higher D-dimer and shorter activated partial thromboplastin time than healthy individuals, DR patients without DR (NDR), and with non-PDR (NPDR), indicating higher level hypercoagulable state in PDR patients (13, 14). In recent years, growing consensus is emerging that the coagulation–fibrinolytic system not only directly results in thrombosis and hemostasis but also promotes inflammation and contributes to retinal vascular dysfunction (15). Various studies demonstrated that platelet adhesion to the injured diabetic endothelium may contribute to both ischemia and inflammation in DR (16, 17). Therefore, the interrupted coagulation–fibrinolytic system plays a key role in diabetes-induced vascular injury and the progression from NDR and NPDR to PDR.

A number of studies have listed diabetes as a systemic risk factor for retinal vein occlusion (RVO) (18–21). Due to the existence of confounding factors between RVO and DR, it has not been proved whether DR itself is an independent risk factor for RVO. However, several studies have indicated mechanical compression of the retinal veins may result in turbulent blood, causing injury to the venous endothelium that leads to venous occlusion (19, 22–25). Therefore, it is speculated that hemostasis within the fibrovascular membranes in PDR may also contribute to thrombogenesis. It would be interesting and significant to further study the different prevalence of RVO between populations with NDR, NPDR, and PDR, which may clarify the association between thrombogenesis and hemostasis within the fibrovascular membranes.

At present, multiple therapeutic strategies have been focusing on the inhibition of the clotting pathway of blood coagulation cascade or platelet aggregation to relieve the vascular dysfunction in DR (26, 27). According to these two cases, PDR patients with fibrovascular membranes may as well benefit from early relief of vascular traction through fibrovascular membrane delamination. As well, it is also necessary to be on the alert for the signs of RVO both before and after surgery.

Antiphospholipid antibodies (aPLs) are a group of antibodies, including aCL, β2-GPI, and lupus anticoagulant, which are related to autoimmune response to phospholipids and are commonly positive in patients with antiphospholipid syndrome (APS) (28). Studies showed aPLs were more prevalent in patients with RVO, and patients positive for aPLs were more prone to develop transient amaurosis fugax, RVO, retinal artery occlusion, and anterior ischemic optic neuropathy, indicating aPLs as a risk factor of ocular thrombogenesis (29, 30). This also explains the positivity of aPLs in these two patients.

This study has some limitations. First, the number of cases included in this study is relatively small. Second, the predicted factors and clinical characteristics of venous thrombosis have not yet be identified. Expansion of the sample size is necessary to further research into the causes and prognostic implications of this phenomenon in the future.

## Conclusion

In summary, these two cases present some known but uncommon features in DR. Though hemostasis and formation of microthrombi in retinal vascular are known to be typical alterations of DR, it is not easy to capture the live image of this phenomenon. These two cases probably provided the first visual evidence of retinal venous thrombogenesis and thrombus movement, which facilitates a better understanding of DR. Early fibrovascular membrane delamination in PDR patients with TRD may acquire better outcomes, as indicated by the different FFA characteristics postoperatively in the two cases.

## Data availability statement

The original contributions presented in the study are included in the article/Supplementary material, further inquiries can be directed to the corresponding author.

## Ethics statement

The studies involving humans were approved by the Medical Ethics Committee of the Second Affiliated Hospital of Zhejiang University School of Medicine, Hangzhou, China. The studies were conducted in accordance with the local legislation and institutional requirements. The participants provided their written informed consent to participate in this study. Written informed consent was obtained from the individual(s) for the publication of any potentially identifiable images or data included in this article.

## Author contributions

DL: Conceptualization, Data curation, Investigation, Methodology, Writing – original draft. HL: Investigation, Methodology, Writing – review & editing. YF: Investigation, Methodology, Writing – review & editing. YW: Conceptualization, Formal analysis, Funding acquisition, Investigation, Methodology, Project administration, Supervision, Writing – review & editing.

## References

[1] Chen Z Chen B Hu P Liu H Zheng D. A preliminary observation on rod cell photobiomodulation in treating diabetic macular edema. Adv Ophthalmol Pract Res. (2022) 2:100051. doi: 10.1016/j.aopr.2022.100051, PMID: 37846386 PMC10577862

[2] Antonetti DA Klein R Gardner TW. Diabetic retinopathy. N Engl J Med. (2012) 366:1227–39. doi: 10.1056/NEJMra1005073 22455417

[3] Aiello LP Avery RL Arrigg PG Keyt BA Jampel HD Shah ST. Vascular endothelial growth factor in ocular fluid of patients with diabetic retinopathy and other retinal disorders. N Engl J Med. (1994) 331:1480–7. doi: 10.1056/NEJM199412013312203, PMID: 7526212

[4] Alkarithi G Duval C Shi Y Macrae FL Ariens RAS. Thrombus structural composition in cardiovascular disease. Arterioscler Thromb Vasc Biol. (2021) 41:2370–83. doi: 10.1161/ATVBAHA.120.315754, PMID: 34261330 PMC8384252

[5] Boeri D Maiello M Lorenzi M. Increased prevalence of microthromboses in retinal capillaries of diabetic individuals. Diabetes. (2001) 50:1432–9. doi: 10.2337/diabetes.50.6.1432, PMID: 11375345

[6] Ceglowska K Nowomiejska K Kiszka A Koss MJ Maciejewski R Rejdak R. Bilateral macular Roth spots as a manifestation of subacute endocarditis. Case Rep Ophthalmol Med. (2015) 2015:493947:1–3. doi: 10.1155/2015/493947, PMID: 26839725 PMC4709653

[7] Ruddy SM Bergstrom R Tivakaran VS. Roth spots. Treasure Island, FL: Stat Pearls (2024). 29494053

[8] Cheung N Mitchell P Wong TY. Diabetic retinopathy. Lancet. (2010) 376:124–36. doi: 10.1016/S0140-6736(09)62124-3 20580421

[9] Zhao H Zhang LD Liu LF Li CQ Song WL Pang YY. Blood levels of glycated hemoglobin, D-dimer, and fibrinogen in diabetic retinopathy. Diabetes Metab Syndr Obes. (2021) 14:2483–8. doi: 10.2147/DMSO.S309068, PMID: 34103957 PMC8180300

[10] Behl T Velpandian T Kotwani A. Role of altered coagulation-fibrinolytic system in the pathophysiology of diabetic retinopathy. Vasc Pharmacol. (2017) 92:1–5. doi: 10.1016/j.vph.2017.03.005 28366840

[11] Yamashiro K Tsujikawa A Ishida S Usui T Kaji Y Honda Y. Platelets accumulate in the diabetic retinal vasculature following endothelial death and suppress blood-retinal barrier breakdown. Am J Pathol. (2003) 163:253–9. doi: 10.1016/S0002-9440(10)63648-6, PMID: 12819029 PMC1868165

[12] Fujisawa T Ikegami H Yamato E Kawaguchi Y Ueda H Shintani M. Association of plasma fibrinogen level and blood pressure with diabetic retinopathy, and renal complications associated with proliferative diabetic retinopathy, in type 2 diabetes mellitus. Diabet Med. (1999) 16:522–6. doi: 10.1046/j.1464-5491.1999.00111.x, PMID: 10391402

[13] Su Y Chen J Dong Z Zhang Y Ma R Kou J. Procoagulant activity of blood and endothelial cells via phosphatidylserine exposure and microparticle delivery in patients with diabetic retinopathy. Cell Physiol Biochem. (2018) 45:2411–20. doi: 10.1159/000488228, PMID: 29554658

[14] Trope GE Lowe GD Ghafour IM Foulds WS Forbes CD. Blood viscosity in proliferative diabetic retinopathy and complicated retinal vein thrombosis. Trans Ophthalmol Soc U K. (1983) 103:108–10. 6197788

[15] Murugesan N Ustunkaya T Feener EP. Thrombosis and hemorrhage in diabetic retinopathy: a perspective from an inflammatory standpoint. Semin Thromb Hemost. (2015) 41:659–64. doi: 10.1055/s-0035-1556731, PMID: 26305236 PMC4765320

[16] Vinik AI Erbas T Park TS Nolan R Pittenger GL. Platelet dysfunction in type 2 diabetes. Diabetes Care. (2001) 24:1476–85. doi: 10.2337/diacare.24.8.1476 11473089

[17] Matsubara Y Murata M Maruyama T Handa M Yamagata N Watanabe G. Association between diabetic retinopathy and genetic variations in alpha2beta1 integrin, a platelet receptor for collagen. Blood. (2000) 95:1560–4. doi: 10.1182/blood.V95.5.1560.005k43_1560_1564, PMID: 10688808

[18] O’Mahoney PR Wong DT Ray JG. Retinal vein occlusion and traditional risk factors for atherosclerosis. Arch Ophthalmol. (2008) 126:692–9. doi: 10.1001/archopht.126.5.692, PMID: 18474782

[19] Rehak M Wiedemann P. Retinal vein thrombosis: pathogenesis and management. J Thromb Haemost. (2010) 8:1886–94. doi: 10.1111/j.1538-7836.2010.03909.x 20492457

[20] Khayat M Williams M Lois N. Ischemic retinal vein occlusion: characterizing the more severe spectrum of retinal vein occlusion. Surv Ophthalmol. (2018) 63:816–50. doi: 10.1016/j.survophthal.2018.04.005, PMID: 29705175

[21] Marcinkowska A Cisiecki S Rozalski M. Platelet and thrombophilia-related risk factors of retinal vein occlusion. J Clin Med. (2021) 10:3080. doi: 10.3390/jcm10143080, PMID: 34300244 PMC8306401

[22] Yau JW Lee P Wong TY Best J Jenkins A. Retinal vein occlusion: an approach to diagnosis, systemic risk factors and management. Intern Med J. (2008) 38:904–10. doi: 10.1111/j.1445-5994.2008.01720.x, PMID: 19120547

[23] Jefferies P Clemett R Day T. An anatomical study of retinal arteriovenous crossings and their role in the pathogenesis of retinal branch vein occlusions. Aust N Z J Ophthalmol. (1993) 21:213–7. doi: 10.1111/j.1442-9071.1993.tb00959.x 8148137

[24] Frangieh GT Green WR Barraquer-Somers E Finkelstein D. Histopathologic study of nine branch retinal vein occlusions. Arch Ophthalmol. (1982) 100:1132–40. doi: 10.1001/archopht.1982.01030040110020, PMID: 6178389

[25] Zhao J Sastry SM Sperduto RD Chew EY Remaley NA. Arteriovenous crossing patterns in branch retinal vein occlusion. The eye disease case-control study group. Ophthalmology. (1993) 100:423–8. doi: 10.1016/s0161-6420(93)31633-7, PMID: 8460014

[26] Tang Z Zhao M Li C Wang Y Peng S. Polyaspartoyl.l-arginine inhibits platelet aggregation through stimulation of NO release from endothelial cells. Eur J Pharmacol. (2008) 588:41–6. doi: 10.1016/j.ejphar.2008.04.012, PMID: 18457831

[27] Qian J Jiang YR. Decreased prothrombin time after intravitreal bevacizumab in the early period in patients with proliferative diabetic retinopathy. Acta Ophthalmol. (2011) 89:e332–5. doi: 10.1111/j.1755-3768.2011.02151.x, PMID: 21470390

[28] Lima Cabrita FV Foster CS. Anticardiolipin antibodies and ocular disease. Ocul Immunol Inflamm. (2005) 13:265–70. doi: 10.1080/09273940490912434 16159716

[29] Suvajac G Stojanovich L Milenkovich S. Ocular manifestations in antiphospholipid syndrome. Autoimmun Rev. (2007) 6:409–14. doi: 10.1016/j.autrev.2006.11.005 17537387

[30] Hernandez JL Sanles I Perez-Montes R Martinez-Taboada VM Olmos JM Salmon Z. Antiphospholipid syndrome and antiphospholipid antibody profile in patients with retinal vein occlusion. Thromb Res. (2020) 190:63–8. doi: 10.1016/j.thromres.2020.04.005, PMID: 32311631

