# Supplementary material for: Case reports: Intraoperative migratory retinal venous thrombus in proliferative diabetic retinopathy
Source: Front Med (Lausanne). 2024 Sep 9;11:1372831. doi: 10.3389/fmed.2024.1372831 (PMC11417017; doi:10.3389/fmed.2024.1372831)
Supplement: Supplementary file 1 [file Data_Sheet_1.DOCX]

**Supplementary material**

**Video S1.** **Intraoperative observation of migratory retinal venous thrombus in Patient 1.**

**Video S2. Intraoperative observation of migratory retinal venous thrombus in Patient 2.**

**Video S3. Intraoperative observation of venous blood flow interruption in Patient 2.**

**Video S4. Arterial and arteriovenous phases of FFA of Patient 1 at 1 months after surgery.**

**Video S5. Venous phase of FFA of Patient 1 at 1 months after surgery.**

**Video S6. Arterial and arteriovenous phases of FFA of Patient 2 at 1 months after surgery.**

**Video S7. Venous phase of FFA of Patient 2 at 1 months after surgery.**

**Video S1.** **Intraoperative observation of migratory retinal venous thrombus in Patient 1.** Rapid movement of tiny white thrombi was observed in the superotemporal branch of the central retinal vein at the time of fibrovascular membrane delamination. The thrombi moved from the site of delamination towards the optic disc when the surgeon pulled the retinal vein during the process of delamination.

**Video S2. Intraoperative observation of migratory retinal venous thrombus in Patient 2.** White migratory thrombi with similar diameter to the vessel were observed in the superotemporal branch of the central retinal vein during fibrovascular membrane delamination.

**Video S3. Intraoperative observation of venous blood flow interruption in Patient 2.** Interruption of blood flow was observed in the superotemporal branch of the central retinal vein with evident segmental whitening of the vessel, which appeared during the process of membrane delamination.

**Video S4. Arterial and arteriovenous phases of FFA of Patient 1 at 1 months after surgery.** Arteriovenous phase of FFA showed normal venous filling without embolus sign.

**Video S5. Venous phase of FFA of Patient 1 at 1 months after surgery.** Venous phase of FFA showed normal venous filling without embolus sign.

**Video S6. Arterial and arteriovenous phases of FFA of Patient 2 at 1 months after surgery.** Arteriovenous phase of FFA showed delayed venous filling without embolus sign.

**Video S7. Venous phase of FFA of Patient 2 at 1 months after surgery.** Venous phase of FFA showed no embolus sign.
